# Ultra-Sensitive Mutation Detection Technology in Myeloid Neoplasms: New Tools for Patient Monitoring

**DOI:** 10.3390/jcm15030998

**Published:** 2026-01-26

**Authors:** Alessandro Ferrando, Valentina Bonuomo, Arianna Savi, Daniela Cilloni

**Affiliations:** Department of Clinical and Biological Sciences, University of Turin, 10124 Orbassano, Italy; alessandro.ferrando@unito.it (A.F.); valentina.bonuomo@unito.it (V.B.); arianna.savi@unito.it (A.S.)

**Keywords:** myeloid neoplasms, patient monitoring, diagnosis, sensitivity, personalized medicine

## Abstract

The clinical management of myeloid neoplasms increasingly relies on the accurate detection and longitudinal monitoring of disease-defining genetic alterations. Many clinically relevant mutations are often present at very low variant allele frequencies, below the detection limits of conventional approaches routinely used in diagnostic workflows. In recent years, a growing number of ultra-sensitive molecular technologies have been developed to overcome these limitations, enabling the detection of rare variants with unprecedented precision, offering complementary strengths in terms of sensitivity, quantification, throughput, and clinical applicability. This review provides a comprehensive overview of established and emerging ultra-sensitive technologies for the diagnosis and molecular monitoring of myeloid neoplasms, discussing their technical principles, advantages, and limitations.

## 1. Introduction

Myeloid neoplasms comprise a heterogeneous group of clonal hematopoietic disorders, including but not limited to acute myeloid leukemia (AML), myelodysplastic syndromes (MDSs), and myeloproliferative neoplasms (MPNs), which arise from the accumulation of genetic and epigenetic alterations affecting hematopoietic stem and progenitor cells [1,2,3]. Accurate diagnosis, prognostic stratification, and therapeutic monitoring are essential to ensure patient treatment decisions, therapy efficacy, and residual disease monitoring and require an integrative, multimodal approach encompassing morphologic, immunophenotypic, cytogenetic, and molecular data [4,5,6]. Historically, the classification of myeloid malignancies relied mainly on morphology and cytochemistry [7]. However, the incorporation of immunophenotyping, chromosomal banding analysis, and molecular genetics has revolutionized clinical practice, leading to the identification of recurrent cytogenetic and molecular abnormalities that define specific disease entities, as reported in the current 5th edition of the World Health Organization (WHO) and International Consensus Classification (ICC) systems [4,5]. Advances in molecular technology have progressively enhanced the sensitivity, resolution, and throughput of diagnostic and monitoring assays, transitioning from single-gene analyses to high-throughput, multiplexed platforms capable of comprehensively characterizing the genomic landscape of individual patients. These innovations have profoundly improved diagnostic accuracy, risk stratification, and therapeutic decision-making, ultimately contributing to better patient outcomes [8]. Nevertheless, significant challenges remain unsolved in terms of limits of detection, throughput, and turnaround time. Emerging technologies hold the promise of overcoming current technical limitations by offering deeper insights into disease biology, more precise therapeutic tailoring, and improved strategies for longitudinal patient monitoring.

In this review, we aim to provide clinicians and laboratory specialists with a comprehensive overview of the state-of-the-art diagnostic and monitoring techniques for myeloid neoplasms, together with the emerging technological innovations that are poised to shape the next generation of clinical assays. These emerging platforms represent an evolution of established approaches, often integrating complementary analytical features, such as increased sensitivity, higher multiplexing capacity, and improved scalability, to achieve unprecedented depth of detection and broader applicability across disease contexts. By outlining both present and future tools, we intend to offer a forward-looking perspective on how diagnostic technologies are expected to evolve and how these advancements may soon translate into more refined, personalized management strategies for patients with myeloid neoplasms.

## 2. Integration of Molecular Diagnostics Across the Spectrum of Myeloid Neoplasms

The diagnosis and management of myeloid neoplasms currently rely on a multilayered diagnostic paradigm integrating cytomorphology, immunophenotyping, cytogenetics, and molecular genetics. Each technique contributes complementary information to the overall diagnostic process:

- **Next-generation sequencing (NGS)** enables comprehensive genomic profiling, allowing simultaneous detection of single-nucleotide variants, indels, and copy-number alterations for precise diagnostic categorization and targeted therapy selection.
- **Flow cytometry** characterizes clonal immunophenotypes and enables measurable residual disease (MRD) detection with high sensitivity.
- **Conventional cytogenetics** provides a global chromosomal overview for risk classification and identification of recurrent structural abnormalities.
- **Sanger sequencing** and **RT-PCR** ensure high-fidelity detection of defined mutations and fusion transcripts essential for diagnosis and therapeutic monitoring.

This integrative framework has transformed clinical hematology from morphology-driven to genomics-informed medicine, allowing refined risk stratification, personalized treatment, and precise disease monitoring. Understanding the fundamental principles, advantages, and limitations of each technique is essential to guide its appropriate application and to drive its continuous optimization and improvement.

Each technique, however, presents peculiar requirements, including sample requirements, technical competencies, analytical sensitivity, turnaround time, cost, and interpretative complexity, which determine its optimal use in the diagnostic workflow. Table 1 summarizes the main methodological features, clinical applications, and limitations of these diagnostic tools as currently applied to myeloid neoplasms.

### 2.1. Next-Generation Sequencing

The introduction of next-generation sequencing in the mid-2000s marked the beginning of a new era of genomic precision medicine in hematology [19]. By enabling massively parallel sequencing of DNA or RNA, NGS allows simultaneous identification of single-nucleotide variants, insertions/deletions, copy-number alterations, and gene fusions within a single assay. In recent years, targeted myeloid gene panels have become standard in the diagnostic workflow for AML, MDS, and MPN [9,11], enabling the detection of dozens of mutations simultaneously, leading to the identification of molecularly defined patient subgroups with established prognostic and therapeutic relevance, as reflected in the most recent ELN 2022 risk classification [20,21,22]. The analytical sensitivity of diagnostic NGS typically ranges from 1 to 2% variant allele frequency (VAF), while recent advanced versions can reach sensitivities of 0.1% or lower, supporting its future use also for MRD monitoring [23]. Despite its transformative potential, NGS still faces several practical and analytical challenges, including high costs, the need for specialized bioinformatics pipelines, low VAF sensitivity, and the ongoing difficulty of interpreting variants of uncertain significance (VUS).

In particular, the progressive increase in analytical sensitivity offered by NGS approaches also raises important challenges in clinical interpretation, as clonal hematopoiesis (CH) detection is becoming increasingly common due to the ubiquitous use of somatic and germline sequencing in clinical practice. Clonal hematopoiesis of indeterminate potential (CHIP) is defined by the presence of somatic pathogenic variants in driver oncogenes, present at a VAF ≥ 2% in the absence of cytopenias, cytosis, or BM dysplasia [24]. By middle age, most individuals have low VAF CH variants [25]. Longitudinal studies have demonstrated that most of these variants largely remain static or even shrink over time, suggesting that only a subset may be clinically relevant later in life.

In patients with myeloid leukemia, the identification of mutations commonly associated with clonal hematopoiesis, such as DNMT3A, TET2, and ASXL1, may either indicate an ancestral lesion shared with the leukemic clone or, alternatively, a parallel and biologically independent clonal population with uncertain clinical significance [24]. Single-cell-based analyses performed on leukemic blasts can, in principle, help disentangle these scenarios by defining clonal hierarchies and mutation co-occurrence at the cellular level.

For MRD purposes, mutation selection should preferentially focus on leukemia-defining or progression-associated variants, while persistent founder-clone mutations associated with clonal hematopoiesis should be interpreted with caution and generally avoided as sole MRD markers.

Despite these clinical challenges, NGS has become the cornerstone of modern molecular hematology, serving as a foundation for molecular diagnosis and an individualized approach to disease management.

### 2.2. Flow Cytometry

Flow cytometry has been a central diagnostic tool in hematology since the late 1970s, when fluorescence-activated cell sorting (FACS) was introduced to characterize hematopoietic populations at the single-cell level [26]. Over the following decades, the technique evolved into multiparametric flow cytometry (MFC), capable of detecting more than 20 surface or cytoplasmic antigens simultaneously. The method relies on the analysis of light scatter and fluorescence emitted by antibody-conjugated fluorochromes as cells pass through a laser beam. Bone marrow aspirates and peripheral blood are the primary specimens used, and proper handling is essential to preserve cell viability and signal accuracy [12]. Flow cytometry is indispensable for the diagnosis, classification, and monitoring of AML, MDS, and, to a lesser extent, MPN. In AML, it defines aberrant antigen expression patterns that support lineage assignment and disease classification, while in MDS, it aids in distinguishing clonal dysplasia from reactive changes [27,28]. In MPNs such as primary myelofibrosis (PMF), flow cytometry can provide supportive evidence of clonality through abnormal myeloid maturation patterns [29]. Beyond technological innovation, a major effort has been directed toward standardization and harmonization of MRD assessment across centers. Initiatives such as the European LeukemiaNet (ELN) MRD Working Party and the EuroFlow consortium have developed harmonized antibody panels, reference gating strategies, and analytical algorithms for reproducible quantification of residual disease [30,31] and enabled MRD detection at levels below 10^−4^. These standards aim to ensure comparability between laboratories and to integrate MFC-derived MRD results with molecular and genomic data for truly multilayered disease monitoring. Despite high sensitivity, short turnaround time (TAT), cost-effectiveness, and the possibility to discriminate between living and dead cells [32], flow cytometry is operator-dependent, requiring expert interpretation and harmonized panels, resulting in a laborious but indispensable component of diagnostic and prognostic assessment in myeloid malignancies.

### 2.3. Conventional Cytogenetics (Karyotyping)

The field of hematologic cytogenetics originated with the discovery of the Philadelphia chromosome (t(9;22)) in CML, providing the first evidence that cancer is driven by genetic abnormalities [33,34]. Since then, chromosomal banding analysis has become a diagnostic mainstay in myeloid malignancies. Conventional karyotyping analyzes metaphase chromosomes from cultured bone marrow cells to identify numerical and structural chromosomal abnormalities with a resolution of approximately 5–10 Mb [14] and is indispensable for AML, MDS, MPN, and CML, as it drives both classification and prognostication. For instance, t(8;21), inv(16), and t(15;17) define favorable AML subgroups, whereas complex or monosomal karyotypes confer poor prognosis [4,22]. In MDS, cytogenetic abnormalities are integral to the Revised International Prognostic Scoring System (IPSS-R), which allows for the identification of an MDS entity defined by the presence of the 5q abnormality, when loss of the long arm of chromosome 5 is detected (MDS5q-) [5]. In MPN, they provide additional prognostic information, particularly in PMF [21]. The major limitations of karyotyping include its reliance on dividing cells, moderate resolution, and time-consuming nature. Complementary techniques such as FISH or array-based comparative genomic hybridization (aCGH) can enhance the detection of cryptic or submicroscopic lesions. Despite these challenges, cytogenetics remains a cornerstone of myeloid neoplasm diagnostics due to its genome-wide, unbiased, and knowledge-free perspective and historical continuity in disease classification.

### 2.4. Sanger Sequencing

The Sanger sequencing method, developed in 1977, revolutionized genetics by enabling direct reading of nucleotide sequences [35]. In hematology, Sanger sequencing remains valuable for targeted mutation analysis in diseases where specific variants have diagnostic or prognostic significance. It is routinely applied to detect NPM1 [36], CEBPA [37], FLT3 [38], and IDH1/2 [39] mutations in AML, as well as CSF3R mutations in chronic neutrophilic leukemia (CNL) [40], and is often employed for CALR mutations, particularly type 1 and type 2 variants, in MPN [41]. Sanger sequencing also serves to confirm mutations initially identified by NGS, given its high accuracy and straightforward interpretation. The method’s limitations (sensitivity around 15–20% VAF, low throughput, and relatively high cost per gene) restrict its use in routine large-scale screening [15], limiting its applicability as a confirmatory or validation tool in contemporary laboratories.

### 2.5. PCR-Based Methods

Reverse transcription/real-time PCR (RT-PCR), allele-specific oligonucleotide quantitative PCR (ASO-qPCR), loop-mediated isothermal amplification PCR (LAMP), and capillary electrophoresis-based PCR (CE-PCR) represent fundamental milestones in molecular hematology, enabling the sensitive and specific detection of gene fusions, mutations, and quantitative transcript analysis [42]. In AML, molecular assays are now integral components of disease diagnosis, risk stratification (as defined by the European LeukemiaNet model), and MRD monitoring [9,22]. In particular, many assays have become the gold standard for the molecular diagnosis and monitoring of several hematologic malignancies, including chronic myeloid leukemia (canonical BCR–ABL1 and atypical fusion transcripts) [16,43], acute promyelocytic leukemia (PML–RARA) [18], myeloproliferative neoplasms (JAK2 V617F) [44], myelodysplastic syndromes (SF3B1) [45], mastocytosis (KIT D816V) [46], and core-binding factor AML (RUNX1–RUNX1T1 and CBFB–MYH11) [47], as well as canonical NPM1-mutated AML (NPM1c^+^) [48].

A paradigmatic limitation of this is the detection of FLT3 mutations, particularly the internal tandem duplication (FLT3-ITD) within the juxtamembrane domain. Unlike the FLT3 tyrosine kinase domain (TKD) mutations, most commonly a point mutation at codon D835, FLT3-ITD results from insertions of variable length and position, generating patient-specific mutations. These characteristics preclude standard RT-PCR detection, as no common primer-binding region exists. Instead, fragment analysis by capillary electrophoresis (CE-PCR) allows qualitative identification of ITDs in a sequence-independent manner and permits quantification of the mutant-to-wild-type allelic ratio, a crucial prognostic parameter incorporated into the ELN 2022 risk classification [22]. The major limitation of CE-PCR is its low sensitivity of approximately 1–5% mutant allele burden, too low for MRD assessment. Many attempts have been made to overcome CE-PCR limitations, but technical challenges are still being addressed [49,50]. More recently, amplicon-based NGS has been developed for FLT3 ITD-based MRD detection [51]. Thanks to the combined use of quantitative PCR and high-sensitivity NGS, the role of FLT3 as a reliable marker of MRD in acute myeloid leukemia has been more precisely defined, clarifying the clinical significance of MRD positivity after completion of induction as well as before and after allogeneic stem-cell transplantation. Evidence from clinical trials with FLT3 inhibitors [52,53,54] consistently shows that persistence of FLT3-based MRD is associated with worse prognosis, higher risk of relapse, and reduced overall survival, thereby reinforcing the importance of FLT3-ITD molecular monitoring to guide therapeutic decisions.

Another key point relevant for laboratory practice is clonal evolution across the disease course: most patients who are FLT3-ITD-positive at diagnosis remain positive at relapse, often with higher allelic burden, but a meaningful fraction of cases gain or lose FLT3 lesions at relapse, supporting repeat FLT3 testing at relapse [55]. In a significant proportion of patients who are FLT3-ITD-negative at diagnosis and subsequently relapse as FLT3-ITD-positive, a small FLT3-ITD clone was already present at diagnosis but below the sensitivity threshold of the detection method used [56]. Moreover, it has been demonstrated that the presence and number of FLT3-ITD microclones at diagnosis or at relapse carry independent adverse prognostic significance, being associated with inferior survival outcomes [57]. Although PCR-NGS provides outstanding sensitivity and accuracy, it remains a complex, labor-intensive, and costly technique that is not accessible to most laboratories; as a result, in many countries, FLT3-based MRD monitoring is still not realistically feasible.

Taken together, these PCR-based approaches remain indispensable tools in modern molecular hematology, offering rapid, sensitive (up to 10^−4^), and cost-effective assessment of well-defined genetic lesions that guide diagnosis, prognosis, and therapeutic monitoring. Despite their inherent limitations, such as dependency on known targets, variable sensitivity across techniques, and challenges in detecting complex or unstable mutations, they continue to form the backbone of routine clinical testing. As newer high-sensitivity and high-throughput technologies emerge, PCR methods retain a complementary role, providing robust, standardized assays that anchor molecular workflows and ensure reliable real-time decision-making in the management of AML and related myeloid neoplasms.

### 2.6. Practical Considerations for the Clinical Use of MRD Assessment Techniques

From a practical standpoint, the clinical use of ultra-sensitive molecular monitoring requires an integrated approach. Peripheral blood (PB) may be appropriate for longitudinal surveillance in selected settings, particularly when highly sensitive assays are employed and disease burden is expected to be low. However, bone marrow (BM) assessment remains essential in cases of suspected relapse, discordant results, or when molecular findings carry potential therapeutic implications. In the presence of discordant flow cytometry and molecular MRD results, clinicians should consider the biological plausibility of the detected variants, their known association with CHIP, and the temporal dynamics of clonal evolution. Integration with morphologic, immunophenotypic, and clinical data remains essential to avoid overinterpretation of ultra-low-level molecular signals.

In clinical practice, the use of MRD assessment methods is reserved for diseases such as chronic myeloid leukemia and acute myeloid leukemia.

In chronic myeloid leukemia, MRD evaluation is mainly based on RT-PCR for the detection and quantification of the BCR-ABL1 transcript in both peripheral blood and bone marrow [58]. Assessment using peripheral blood is routinely applied for standard monitoring of molecular response, whereas bone marrow analysis is preferred in selected cases in which a more in-depth evaluation is required or when there is suspicion of disease progression or transformation [59].

**Figure 1:**
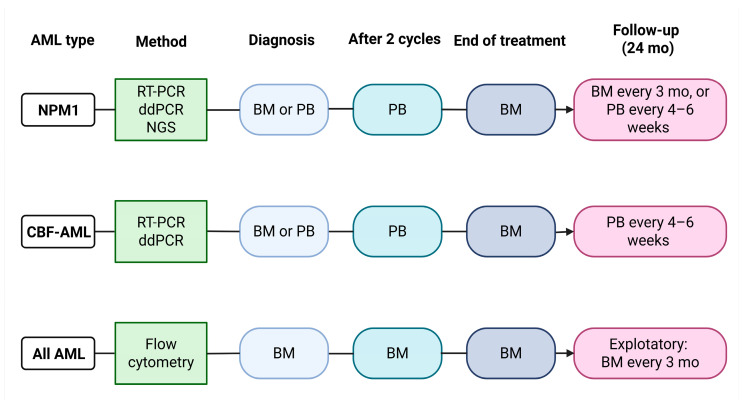
MRD assessment in AML. Schematic representation of the algorithm of MRD assessment in AML, with reported AML subtypes, molecular methodologies, time points, and biological materials used for MRD assessment. BM = bone marrow; PB = peripheral blood. Adapted from Döhner et al. [22]. Created in BioRender. Pergolizzi, B. (2026) https://BioRender.com/p9iky9t.

In the context of acute myeloid leukemia, the assessment of MRD is performed using RT-qPCR when a stable molecular marker is available, such as the NPM1 mutation or core-binding factor (CBF) fusion transcripts (RUNX1–RUNX1T1 and CBFB–MYH11). In the absence of specific molecular abnormalities suitable for molecular monitoring, MRD evaluation can be carried out by flow cytometry, through the identification of leukemia-associated aberrant immunophenotypes (LAIP), or by using the different-from-normal (DFN) approach [60]. According to the 2022 ELN guidelines, MRD assessment should be performed for the evaluation of treatment response (post-induction and after two cycles of treatment) and for disease monitoring once a remission has been achieved. This assessment is primarily carried out on bone marrow samples, except for molecular MRD monitoring based on NPM1 mutations or CBF fusion transcripts, which can be performed on peripheral blood to track potential relapse once bone marrow MRD negativity has been achieved post-treatment [22]. A schematic representation of MRD monitoring in AML is presented in Figure 1. 

## 3. Limitations of Current Diagnostic Technologies and the Need for Enhanced Sensitivity

Despite these major advances, current diagnostic technologies exhibit inherent limitations that constrain their sensitivity, dynamic range, and scope of genomic coverage.

Traditional approaches such as Sanger sequencing and RT-PCR are targeted by design, relying on prior knowledge of specific mutations or fusion transcripts; consequently, novel or rare genetic events remain undetected. Even NGS, though more comprehensive, has a practical detection limit (typically around 1–3% VAF in standard clinical panels), which may result in false negatives in samples with low tumor burden or in the context of clonal hematopoiesis [61]. Similarly, conventional cytogenetics lacks the resolution to identify submicroscopic alterations, and flow cytometry, while highly sensitive for immunophenotypic abnormalities, cannot directly capture molecular heterogeneity.

**Figure 2:**
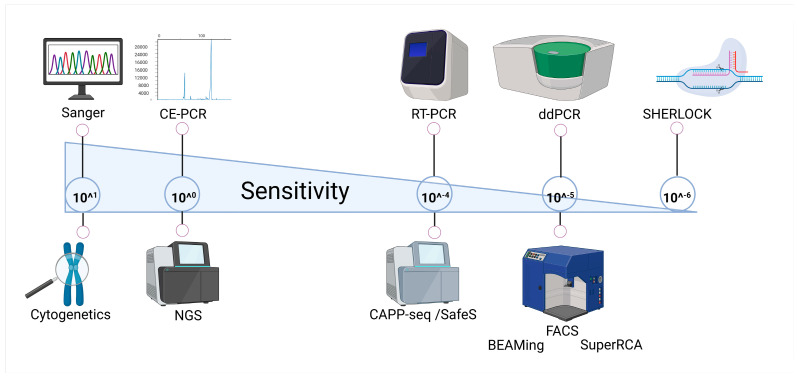
**Sensitivity spectrum of molecular techniques.** Schematic representation of commonly used and emerging molecular technologies positioned along a gradient of analytical sensitivity. Created in BioRender. Pergolizzi, B. (2026) https://BioRender.com/6urvrta.

These limitations underscore the need for next-generation, rapid, and ultra-sensitive approaches capable of detecting rare variants, structural abnormalities, and clone evolution across the myeloid genome and transcriptome. Technologies such as digital droplet PCR (ddPCR), BEAMing, SuperRCA, CAPP-Seq, Safe-SeqS, and the novel SHERLOCK technique are currently under validation for clinical use. They promise to bridge the gap between quantitative sensitivity and comprehensive coverage, offering both molecular depth and unbiased discovery power, providing a broad coverage of clinical need, ranging from diagnosis to patient monitoring (summarized in Figure 2). Methodological features, clinical applications, and limitations of these techniques are presented in Table 2.

## 4. Ultra-Sensitive Diagnosis with NGS: Towards Personalized Mutational Profiling

The advent of ultra-sensitive targeted next-generation sequencing (ustNGS) techniques has significantly advanced the detection of disease-associated mutations at diagnosis in hematologic malignancies. Compared to conventional NGS or standard molecular assays, these methods offer exceptionally high sensitivity, enabling the identification of low-frequency variants and subclonal populations that would otherwise remain undetectable. While traditional NGS provides broad genomic coverage and the ability to discover novel mutations, its sensitivity is also limited in targeted settings, typically detecting variants only when present at 1–5% VAF. In contrast, ustNGS approaches, such as CAPP-Seq and Safe-SeqS, focus on predefined panels of genomic regions of clinical interest (i.e., recurrent mutation of desired disease). By restricting analysis to these targeted loci, sequencing depth can be increased dramatically, often reaching 10,000×–100,000× coverage, which allows detection of variants at VAFs as low as 0.01% (10^−4^) or even lower.

A key limitation of these targeted approaches is that they are designed to detect known, preselected mutations and, therefore, lack the capacity to identify novel or unexpected genetic alterations. Nonetheless, the substantial gain in sensitivity and quantitative precision provided by these methods makes them invaluable for comprehensive molecular profiling at diagnosis, enabling accurate characterization of the mutational landscape, detection of rare subclones, and informed risk stratification. By combining high sensitivity, absolute quantification, and reproducibility, ustNGS represents a powerful complement to broader genomic profiling, bridging the gap between conventional NGS and clinical needs for precise, early detection of clinically relevant mutations.

### 4.1. CAPP-Seq

CAPP-Seq (cancer personalized profiling by deep sequencing) has emerged as a highly precise and powerful NGS-based technology for detecting rare mutations and disease-associated genetic alterations in patient samples, well-suited for identifying low-frequency variants and tracking clonal evolution with exceptional specificity and sensitivity [71,72]. The method relies on hybrid-capture-based targeted sequencing, in which biotinylated DNA probes are designed to selectively bind genomic regions of interest, allowing for the enrichment of tumor-derived DNA fragments from complex mixtures of circulating cell-free DNA (cfDNA) or genomic DNA. By focusing on a selected panel of genomic regions (ranging from hundreds to a few thousand loci) rather than sequencing the whole genome or exome, CAPP-Seq achieves a substantially higher sequencing depth. Compared to ~1000× coverage typical of conventional targeted NGS, which limits detection to variants with VAF ≥1–5%, CAPP-Seq routinely reaches ultra-deep coverage of 10,000×–100,000× per target, enabling detection of extremely rare variants, offering sensitivity and precision far beyond what is achievable with standard NGS approaches.

This approach achieves remarkable sensitivity, reliably detecting mutations with VAF as low as 0.01%, equivalent to identifying a single mutant molecule among 10,000 wild-type sequences, but even lower detection thresholds have been described in optimized assays. Such precision enables the identification of rare subclonal populations that would be missed by conventional sequencing or standard PCR-based methods, representing a two-log improvement in sensitivity.

Although CAPP-Seq was originally developed for liquid biopsy applications, it has found extensive use in hematologic malignancies. In oncohematology, it has been successfully applied to a variety of B-cell lymphoproliferative disorders, including diffuse large B-cell lymphoma (DLBCL), follicular lymphoma (FL), and post-transplant lymphoproliferative disorder (PTLD). Beyond lymphoma, the technology holds significant promise for myeloid neoplasms, where CAPP-Seq has been explored for genomic profiling, disease monitoring, MRD assessment, and clonal evolution analysis without the need for repeated bone marrow biopsies [11,68,73,74]. By enabling non-invasive, serial monitoring of tumor-specific mutations and providing comprehensive molecular profiling from a simple blood draw, CAPP-Seq offers critical insights into clonal dynamics, treatment response, and early molecular relapse, supporting real-time, evidence-based clinical decision-making and highlighting its potential as a transformative tool and future standard in precision hematology for patient monitoring and disease management.

### 4.2. Safe-SeqS

Safe-SeqS (Safe-Sequencing System) is an ustNGS technique designed to accurately detect rare mutations and minimize sequencing errors, making it particularly valuable for hematologic malignancies [61]. The method is based on the use of unique molecular identifiers (UMIs), which are short, random DNA barcodes attached to each original DNA molecule prior to amplification. This allows every sequencing read to be traced back to its individual template molecule. By grouping reads that share the same UMI and generating a consensus sequence, Safe-SeqS effectively eliminates most PCR and sequencing errors, dramatically increasing specificity and enabling the reliable detection of extremely low-frequency variants [61].

An extension of this concept, often referred to as duplex sequencing, further improves accuracy by independently tagging and sequencing both strands of each DNA molecule. True mutations are confirmed only when they are observed in both complementary strands, whereas errors introduced during PCR or sequencing typically appear on a single strand and can be discarded. This strategy reduces background error rates to as low as 10^−7^–10^−6^, allowing Safe-SeqS to detect mutations with VAF far below the limits of conventional NGS approaches. In optimized duplex sequencing protocols (e.g., use of a targeted panel), detection can reach 10^−4^ or lower, meaning it can identify a single mutant molecule among 10,000 wild-type molecules (0.01%), far surpassing the sensitivity of conventional NGS [75].

Despite its strengths, Safe-SeqS has several limitations. Its targeted nature prevents the discovery of novel or unexpected mutations outside the selected panel. The method requires high-quality DNA and sufficient input, which can be challenging in low-burden or liquid biopsy samples. Ultra-deep sequencing and UMI-based error correction increase cost, complexity, laborious protocol, library prep, and processing time compared to simpler PCR-based assays. Finally, the approach demands specialized bioinformatics pipelines for consensus building and duplex error correction, which may limit adoption in laboratories without dedicated computational resources.

In the context of myeloid neoplasms, Safe-SeqS plays a role in both the detection and monitoring of disease by enabling highly sensitive identification of clinically relevant, low VAF mutations, resulting in an excellent methodology for accurate diagnosis, risk stratification, and MRD assessment [76,77]. While Safe-SeqS is not yet universally incorporated into clinical guidelines, its technical principles underpin many of the advanced NGS assays now validated for clinical use in myeloid neoplasms, resulting in an excellent candidate for future molecular analysis in oncohematology.

## 5. Patient Monitoring: The Need for Fast and Accurate Marker Detection

The advent of molecular techniques has transformed disease monitoring in hematologic malignancies, providing a level of sensitivity, specificity, and clinical utility that far surpasses conventional approaches such as cytogenetics, morphology, or flow cytometry alone [78]. Traditional methods, while still valuable for diagnosis and disease classification, often lack the resolution needed to detect minimal levels of residual disease and may fail to identify early molecular relapse. In contrast, molecular patient monitoring enables the precise detection and quantification of disease-associated genetic markers, allowing clinicians to track clonal dynamics with exceptional accuracy and to make timely, informed therapeutic decisions. A central concept underpinning this approach is minimal residual disease (MRD), a value that represents the presence of a small number of leukemic cells that persist below the threshold of conventional detection methods [79]. MRD assessment has emerged as one of the most powerful prognostic and predictive tools in modern hematology, informing critical aspects of patient management such as risk stratification, treatment intensification or de-escalation, and decisions regarding therapy continuation, discontinuation, or switching [80]. Reliable MRD detection has been associated with improved patient outcomes, including reduced relapse risk and prolonged survival [79]. The conventional definition of MRD negativity is strictly dependent on the analytical method employed, as it is determined by the sensitivity threshold that the technique can guarantee, generally not lower than 10^−3^. This methodological variability has contributed to a progressive redefinition of the term MRD, which is increasingly shifting from “minimal residual disease” to “measurable residual disease,” highlighting that MRD refers to the lowest amount of disease that can be reliably detected with the technology used [30,81]. These clinical demands have driven the development of novel molecular technologies that deliver rapid, sensitive, cost-effective, and highly accurate detection of disease-specific genetic alterations. Among the most widely adopted molecular approaches for disease monitoring are digital droplet PCR (ddPCR), BEAMing, and the more recently developed super rolling circle amplification (SuperRCA). Each of these methods offers unique advantages in terms of sensitivity, quantification, throughput, and practicality, collectively enabling a more refined and personalized approach to patient follow-up in myeloid neoplasms. While RT-PCR remains the most widely implemented technique in routine diagnostic laboratories, ddPCR, BEAMing, and SuperRCA, though currently applied mainly in research contexts or in a limited number of specialized centers, demonstrate superior analytical performance and hold strong potential to become part of future standard-of-care workflows. As these technologies continue to mature, their integration into clinical practice is expected to further enhance molecular monitoring, bridging the gap between diagnostic precision and real-time therapeutic decision-making.

### 5.1. Digital Droplet PCR

Still relying on DNA amplification by PCR, digital droplet PCR (ddPCR) represents a unique alternative to RT-PCR-based methods, enabling ultra-sensitive, precise, and absolute quantification of nucleic acid targets [62,63]. The technique works by partitioning a conventional PCR mixture into tens of thousands of nanoliter-sized oil-emulsion droplets, each acting as an independent reaction microchamber within a single tube. Following thermal cycling, each droplet is individually analyzed to determine whether the target sequence is present or absent, generating a binary digital readout. By distributing target DNA or cDNA molecules such that, on average, a single template is encapsulated per droplet, ddPCR achieves remarkable sensitivity, detecting VAF as low as 0.001% (10^−5^), equivalent to identifying a single mutated molecule among approximately 40,000 wild-type sequences. Importantly, because each droplet functions as a discrete reaction unit, ddPCR inherently provides absolute quantification without the need for external standard curves, relying instead on internal reference controls or housekeeping targets for normalization. Detection relies on sequence-specific fluorescent probes that emit a signal upon incorporation into the amplified product. This feature allows the use of multiplexing (i.e., the detection of multiple targets in a single reaction) by combining distinct primer–probe sets labeled with different fluorophores. This allows parallel detection of up to 16 mutations or fusion events in a single well, significantly reducing both turnaround time and costs, enabling rapid and cost-effective molecular follow-up that can guide clinical decision-making [62].

In the context of myeloid neoplasms, these features make ddPCR a powerful tool for MRD assessment, mutation burden quantification, and treatment response monitoring [82]. ddPCR has also gained relevance in the molecular evaluation of several rare or diagnostically challenging conditions. One notable application is the quantitative assessment of atypical BCR–ABL1 transcripts in CML patients with uncommon fusion variants, for whom conventional RT-PCR assays are often inadequate. Recent work has demonstrated that multiplex ddPCR enables highly sensitive monitoring of these atypical transcripts [83], supporting accurate molecular response assessment and potentially informing TFR strategies even in this subset of CML patients. Additionally, ddPCR has emerged as a sensitive tool for detecting and quantifying low-level KIT D816V mutant alleles, outperforming standard sequencing-based methods and enhancing diagnostic accuracy in disorders such as systemic mastocytosis [84]. Another expanding application is the molecular diagnosis of hereditary alpha-tryptasemia (HαT), where ddPCR-based copy-number analysis of TPSAB1 and related tryptase gene loci enables precise genotyping and facilitates the interpretation of elevated basal serum tryptase levels [85]. Together, these examples underscore the versatility of ddPCR and its growing role beyond classical myeloid neoplasms, particularly in settings requiring high sensitivity and quantitative precision.

### 5.2. BEAMing: Combining PCR Partitioning with Bead-Flow Readout

BEAMing (beads, emulsion, amplification, and magnetics) is an ultra-sensitive, PCR-based technology designed to overcome the technical challenges of detecting rare DNA variants in complex biological samples [66]. The method combines digital PCR principles with flow cytometry to achieve exceptionally high resolution in the detection and quantification of low-frequency mutations. Unlike conventional PCR, which generates a single signal from a bulk reaction, BEAMing partitions the amplification process into millions of individual microreactions, each corresponding to a single DNA molecule. In this approach, individual DNA templates are compartmentalized into water-in-oil emulsions, where each droplet contains one template molecule and a magnetic bead coated with sequence-specific primers. During amplification, each template is clonally amplified and bound to a single bead, resulting in a one-to-one representation of the original DNA molecules in the sample. These beads are subsequently hybridized with fluorescently labeled probes that distinguish mutant from wild-type sequences, and flow cytometry is used to analyze and quantify the resulting populations [67]. BEAMing is capable of analyzing hundreds of millions of individual DNA molecules simultaneously using standard laboratory equipment and achieves a sensitivity of around 0.001% (10^−5^), enabling the detection of extremely rare somatic mutations, such as RAS variants, even at very low allele frequencies [86]. In oncohematology, BEAMing has been successfully applied for the detection and quantification of cell-free tumor DNA (ctDNA), monitoring of minimal residual disease (MRD), and tracking of clonal evolution during and after therapy. Its combination of digital precision, quantitative accuracy, and high sensitivity makes it a powerful tool for molecular disease monitoring and an important complement to emerging technologies such as ddPCR and SuperRCA.

Clinically, BEAMing technology has already been successfully implemented for the detection of RAS mutations in solid tumors, most notably in colorectal cancer, where sensitive identification of *KRAS* and *NRAS* variants is crucial for guiding targeted therapy decisions [86]. Beyond solid tumors, this approach holds significant promise in the field of hematologic malignancies. In particular, BEAMing could be applied to RAS-mutated acute myeloid leukemia for longitudinal patient monitoring and detection of emerging subclonal populations, which are often associated with disease progression or therapeutic resistance [87].

The technology has also been applied to the detection of *IDH1* and *IDH2* mutations in AML, which are among the most frequent and clinically significant genetic alterations in this disease, carrying diagnostic, prognostic, and therapeutic relevance. Recent studies have demonstrated the successful use of BEAMing for molecular monitoring of AML patients harboring *IDH1/2* mutations undergoing targeted therapy with ivosidenib or enasidenib, enabling precise assessment of treatment response and residual disease detection [88,89]. Remarkably, BEAMing achieved a VAF as low as 0.02%, underscoring its potential for highly sensitive, real-time monitoring. These findings highlight BEAMing as a powerful tool for personalized therapeutic management and dynamic response assessment in IDH-mutated AML.

### 5.3. SuperRCA

The FACS-based technique SuperRCA (super rolling circle amplification) represents a unique, next-generation, ultra-sensitive nucleic acid detection technique that enables precise identification and quantification of rare genetic variants at extremely low allele frequencies [64,65]. This technique still relies on nucleic acid amplification for detecting mutation events but combines this approach with the processivity, efficiency, and high-throughput capacity of flow cytometry, reaching sensitivities not obtainable with conventional techniques. The method is based on the principle of isothermal rolling circle amplification (RCA), in which specially designed padlock probes hybridize to a target DNA or cDNA sequence and are ligated to form circular DNA templates. These circular templates then undergo repeated rounds of RCA, producing long, tandem DNA concatemers that serve as amplified detection scaffolds. The resulting amplification products are subsequently hybridized with fluorescently labeled detection probes, generating strong and easily measurable fluorescence signals even from single-molecule starting material. The amplified products are then analyzed using a flow cytometer, which quantifies the fluorescence intensity of individual amplification events, allowing precise discrimination between mutant and wild-type targets and enabling highly sensitive, single-molecule detection. SuperRCA typically uses genomic DNA or cDNA derived from patient samples as input material, enabling the detection of both mutations and fusion transcripts associated with myeloid malignancies. The method achieves exceptional sensitivity, reliably identifying VAF as low as 0.001% (10^−5^), which is the detection of a single mutant molecule among one million wild-type sequences. Results are obtained within 24–36 h, and although the method is more technically complex than conventional PCR, the overall cost per target is relatively low due to the absence of expensive instrumentation and the potential for high-throughput sample processing. A key advantage of SuperRCA is that it provides absolute, quantitative results, as each amplified signal corresponds to an individual target molecule present in the original sample. The high signal-to-noise ratio, combined with the specificity conferred by dual hybridization and ligation steps, allows for accurate discrimination of single-nucleotide variants or rare clones that may remain undetectable with other technologies. Furthermore, SuperRCA can be multiplexed to simultaneously interrogate multiple mutations within a single reaction, facilitating comprehensive molecular profiling in a time- and cost-efficient manner. In the context of myeloid neoplasms, SuperRCA has shown great promise for MRD detection, early relapse prediction, and quantification of persistent leukemic clones. Its ability to detect extremely rare variants months before clinical or hematological relapse makes it a powerful complementary tool to established techniques such as ddPCR and next-generation sequencing, offering a new level of sensitivity and precision in molecular disease monitoring.

Currently, the use of this method is limited to translational clinical studies, but some clinical applications are starting to emerge in the literature, such as the monitoring of IDH1 and IDH2 in AML [90]. The study by Boertjes et al. applied the SuperRCA method to evaluate these mutations in patients in complete remission after the second cycle of intensive chemotherapy. MRD monitoring for IDH1/IDH2 was performed using both NGS and SuperRCA, showing concordance between the two methods in 89% of cases (114 patients) [90]. Among the discordant cases, one patient had MRD undetectable by SuperRCA but positive by NGS, while in five patients, MRD was detectable by SuperRCA but not by NGS. These findings suggest that SuperRCA is a highly sensitive and promising method for MRD monitoring, demonstrating good concordance with NGS and the potential to detect rare clones undetectable by conventional techniques.

### 5.4. Beyond the Future: CRISPR-Mediated Mutation Detection

SHERLOCK (specific high-sensitivity enzymatic reporter unLOCKing) represents a fascinating new class of molecular diagnostics that leverages CRISPR-Cas systems for ultra-sensitive and specific detection of nucleic acids [69]. Originally described by Kellner et al., the method integrates a pre-amplification step, typically recombinase polymerase amplification (RPA) or reverse transcription-RPA, with CRISPR/Cas enzymology to enable highly sensitive detection of DNA or RNA targets. Upon recognition of a specific sequence by a guide RNA-programmed CRISPR effector (such as Cas12 for DNA or Cas13 for RNA), a collateral cleavage activity is triggered, cutting labeled reporter molecules and generating a detectable fluorescent or colorimetric signal. Results can be read within an hour, either via fluorescence or lateral flow assays, making SHERLOCK simple, rapid, and highly portable, resulting in an attractive feature for point-of-care diagnostics or low-resource settings. The platform also supports multiplexing through the use of multiple crRNAs, enabling simultaneous detection of several targets in a single reaction. SHERLOCK’s strength lies in its exquisite specificity and sensitivity, which allow discrimination of single-nucleotide variants. This feature ensures robustness of the system and exquisite detection of even a single molecule of either DNA or RNA in a 1 µl solution (2 aM), making it a promising candidate for ultra-precision diagnostics [70]. Moreover, it retains the possibility for detection of short insertions or deletions within the 20–30 nucleotide window defined by the guide RNA. This could theoretically allow the identification of known fusion breakpoints, such as BCR–ABL or PML–RARA, provided the junction sequence is well characterized, which opens the door to its application in hematologic malignancies. As with basically all the technologies described so far, SHERLOCK suffers from the same limitations, that is, its limited applicability for unknown or highly variable translocation breakpoints, which are common mutations in leukemias. This is due to the fact that precise crRNA–target complementarity is essential for detection. Additionally, SHERLOCK is generally unsuitable for identifying large structural variants (e.g., duplications, deletions, or complex rearrangements) or copy-number alterations, and it does not provide quantitative information on gene dosage. Despite these limitations, SHERLOCK’s rapid turnaround time, portability, and ability to detect mutations at extremely low abundance position it as a promising diagnostic tool for molecular monitoring in hematology. Its flexibility and potential for integration into clinical workflows make SHERLOCK an exciting candidate for future applications in precision hematology and real-time patient management.

## 6. Conclusions

Molecular techniques have profoundly transformed the management of hematological malignancies, playing a central role not only during the diagnostic phase, where they enable the precise identification of underlying genetic alterations, but also in disease monitoring. These approaches allow for a more accurate classification, improved risk stratification, and targeted therapeutic strategies from the earliest stages of myeloid diseases, overcoming the limitations of conventional methods.

MRD assessment has emerged as one of the most powerful prognostic and predictive tools, guiding key decisions regarding treatment management. The ability to detect clonal persistence at submicroscopic levels allows for earlier therapeutic interventions and more effective disease control.

Innovative technologies such as ddPCR, BEAMing, and SuperRCA offer sensitivity, accuracy, and speed far superior to traditional approaches. Although these methods are currently employed mainly in research settings or highly specialized centers, they show strong potential for broader integration into routine diagnostics and clinical monitoring. Their wider implementation could contribute to the standardization and optimization of diagnostic workflows, ensuring greater accuracy in risk stratification and therapeutic decision-making.

In an era of increasingly personalized medicine, the expansion and harmonization of molecular techniques will support more informed, dynamic, and timely management of myeloid neoplasms. The evolution and broader adoption of these tools represent a crucial step toward improving early diagnosis and monitoring, ultimately translating into significant benefits for patient survival and quality of life.

## Figures and Tables

**Table 1:** Comparison of molecular techniques currently used for the detection and monitoring of genetic alterations in hematologic malignancies. The table summarizes sample requirements, analytical sensitivity, turnaround time, relative cost, principal clinical applications, detectable mutations, and key limitations of each method. BM = bone marrow; PB = peripheral blood.

Technique	Sample/ Material	Analytical Sensitivity	Turnaround Time	Relative Cost	Main Clinical Applications	Gene/Mutation Detected	Limitations	Key Ref.
Next Generation Sequencing	DNA/RNA	10^0^ (targeted), <10^−1^ with deep coverage	14–30 days	High	Comprehensive mutational profiling, prognostic stratification, detection of co-mutations, MRD tracking	All genomes/panels of commonly mutated genes (targeted)	Expensive, complex bioinformatics may miss structural variants	[9,10,11]
Flow Cytometry	Fresh BM/PB (EDTA)	10^−5^	Same day to 48 h	Moderate	Immunophenotypic diagnosis, lineage assignment, MRD monitoring	CD34, CD117, CD45, HLA-DR, CD33, CD123, CD9, CD56	Requires viable cells, operator-dependent, antigen variability	[10,12,13]
Conventional Cytogenetics (Karyotyping)	BM (dividing cells)	~10^1^ (requires ≥2 abnormal metaphases on 20 metaphases)	7–14 days	Moderate	Cytogenetic classification, risk stratification, detection of large chromosomal abnormalities	Cytogenetic abnormalities	Requires cell culture, low resolution, failure in hypocellular samples	[14]
Sanger Sequencing	DNA or RNA	~10^1^	3–5 days	Low–moderate	Detection of single known mutations, validation of NGS results	NPM1, FLT3, IDH1/2, CSF3R, CALR	Low sensitivity, low throughput, not suitable for MRD	[15]
RT-PCR/ASO-qPCR/LAMP/ CE-PCR	RNA (fusion transcripts) or DNA (indels)	10^−4^/ 10^0^ (CE-PCR)	3–5 days	Moderate	Detection and quantification of fusion transcripts and specific mutations, FLT3-ITD analysis (CE-PCR)	NPM1, BCR-ABL, JAK2, IDH1/2, RUNX1–RUNX1T1 (RT-PCR)/ KIT (ASO-qPCR)/ PML-RARA (LAMP)/ FLT3-ITD (CE-PCR)	Limited to known targets, requires high-quality starting material	[16,17,18]

**Table 2:** Comparison of selected ultra-sensitive molecular techniques used for the detection and monitoring of genetic alterations in hematologic malignancies. The table summarizes sample requirements, analytical sensitivity, turnaround time, relative cost, principal clinical applications, detectable mutations, and key limitations of each method.

Technique	Sample/ Material	Analytical Sensitivity	Turnaround Time	Relative Cost	Main Clinical Applications	Genes/Mutations Detected	Limitations	Key Ref.
ddPCR	DNA or RNA	10^−5^	Same day to 48 h	Moderate	Detection of mutations, MRD monitoring, risk stratification	KIT, BCR-ABL1,	Limited to known targets, high-quality material	[62,63]
superRCA	DNA or RNA	10^−5^	Same day to 48 h	Moderate	Detection of mutations, MRD monitoring, risk stratification	IDH1/2	Limited to known targets, high-quality material	[64,65]
BEAMing	DNA or RNA	10^−5^	Same day to 48 h	Moderate	Detection of mutations, MRD monitoring, risk stratification	N/KRAS, IDH1/2	Limited to known targets, high-quality material	[66,67]
CAPP-seq	DNA or RNA	10^−4^	5–14 days	High	Mutational profiling, prognostic stratification, detection of co-mutations, MRD tracking	All genome/panel of commonly mutated genes (targeted)	Expensive, complex bioinformatics, requires skilled operators, limited to known targets, high-quality material	[68]
Safe-seqS	DNA or RNA	10^−4^	5–14 days	High	Mutational profiling, prognostic stratification, detection of co-mutations, MRD tracking	All genome/panel of commonly mutated genes (targeted)	Expensive, complex bioinformatics, requires skilled operators, limited to known targets, high-quality material	[61]
SHERLOCK	DNA or RNA	10^−6^	Same day to 48 h	Moderate	Detection of mutations, MRD monitoring	BCR-ABL1, PML-RARA	Limited to known targets, high-quality material	[69,70]

## Data Availability

No new data were created or analyzed in this study. Data sharing is not applicable to this article.

## Abbreviations

The following abbreviations are used in this manuscript:

AML	Acute Myeloid Leukemia
MDS	Myelodysplastic Syndrome
CML	Chronic Myeloid Leukemia
MPN	Myeloid Proliferative Neoplasm
CNL	Chronic Neutrophilic Leukemia
MRD	Measurable residual disease
VAF	Variant Allele Frequency
BM	Bone Marrow
PB	Peripheral Blood
ddPCR	Digital Droplet PCR
BEAMing	Beads, Emulsion, Amplification, and Magnetics
SuperRCA	Super Rolling cycle assay
NGS	Next-Generation Sequencing
SHERLOCK	Specific High-Sensitivity Enzymatic Reporter Unlocking
CAPP-seq	Cancer Personalized Profiling by Deep Sequencing
RT-PCR	Real-Time PCR
CE-PCR	Capillary Electrophoresis PCR
ASO-qPCR	Allele-Specific Oligonucleotide Quantitative PCR
LAMP	Loop-Mediated Isothermal Amplification PCR
HαT	Hereditary Alpha Tryptasemy
